# A Brief Look at Hashimoto’s Disease, Adrenal Incidentalomas, Obesity and Insulin Resistance—Could Endocrine Disruptors Be the Other Side of the Same Coin?

**DOI:** 10.3390/medicina59071234

**Published:** 2023-06-30

**Authors:** Katarzyna Gontarz-Nowak, Michał Szklarz, Magdalena Szychlińska, Wojciech Matuszewski, Elżbieta Bandurska-Stankiewicz

**Affiliations:** Department of Internal Medicine, Faculty of Medical Sciences, University of Warmia and Mazury in Olsztyn, 10-719 Olsztyn, Poland

**Keywords:** endocrine disruptors, thyroid autoimmunity, adrenal incidentaloma, insulin resistance, obesity

## Abstract

Hashimoto’s disease (HD) is the most common cause of hypothyroidism in developed countries. The exact pathomechanism behind it has not been clearly established; however, an interplay of genetic susceptibility, environmental triggers (including diet) and epigenetic factors seems to be involved. Among the latter, increasingly more attention has been paid to some hormonally active substances, known as endocrine disruptors, which are commonly used worldwide. HD has become a condition widely reported in the media, acting as a culprit for inexplicable weight gain, chronic fatigue or weakness. Nevertheless, the recognition of HD is undeniably increasing and represents a major public health burden. At the same time, improving access to imaging tests has increased the number of incidentally diagnosed adrenal tumors. Above all, the widespread use of chest computed tomography (CT) due to the COVID-19 pandemic has contributed to frequent incidental detection of adrenal lesions. Fortunately, a vast majority of these findings are asymptomatic benign tumors with no excessive hormonal activity, and therefore, they are defined as adrenal incidentalomas (AIs). Interestingly, recent studies have indicated that patients with AIs are more prone to obesity and insulin resistance. Although mutual relationships between the thyroid and the adrenal glands have been studied widely, still, little is known about the possible pathophysiological associations between thyroid autoimmunity and the occurrence of adrenal incidentalomas. This article presents a brief review of the common endocrine disorders with a special focus on the frequently coexisting insulin resistance and/or obesity. Furthermore, in response to the recent growing interest in endocrine disruptors, with their transgenerational epigenetic effects that influence hormonal system function, a concise overview of the topic has also been included.

## 1. Introduction

### 1.1. Thyroid Autoimmunity

So far, thyroid autoimmunity, mainly referring to Hashimoto’s disease (HD), also known as chronic autoimmune thyroiditis, has been the most common disorder in clinical endocrine practice [1]. According to the literature, in countries where iodine prophylaxis has been introduced, an increase in the incidence of autoimmune thyroid disease has been noted [1]. In fact, the thyroid gland is one of the organs most frequently involved in the autoimmune process [2,3]. Currently, HD is the most common cause of hypothyroidism in developed countries [2,4]. The diagnosis of HD is based on a positive titer of anti-thyroid peroxidase (a-TPO) and anti-thyroglobulin (a-TG) antibodies, often with a concomitant increase in TSH levels and a characteristic hypoechoic image of the thyroid parenchyma on ultrasound [5]. Its clinical symptoms are not limited to the thyroid gland, but also include systemic manifestations related mainly to thyroid hormone deficiency. These include fatigue, weight gain, constipation, increased sensitivity to cold, dry skin, depression, muscle pain, reduced exercise tolerance and irregular menstruation. In some cases, inflammation causes the thyroid to become enlarged, leading to goiter, which, rarely, may cause neck discomfort or difficulty swallowing. Since the thyroid gland is one of the organs most affected by autoimmune processes, many patients with HD seek medical advice on lifestyle changes and dietary modifications to improve and maintain their thyroid function. Although the official guidelines do not indicate any specific dietary treatment in HD, apart from a generally healthy lifestyle, more attention has recently been paid to limiting the use of substances that exert hormonal activity, known as endocrine disruptors (EDs).

According to the definition of the Polish Society of Endocrinology, EDs are exogenous chemical substances, synthetic or natural, which occur in the environment or food and interfere with the endocrine system [6]. According to World Health Organization (WHO), EDs are specific compounds that can cause adverse effects on human bodies, offspring or subpopulations [7]. EDs include various substances, such as dioxins, phthalates, phenols (including the commonly known Bisphenol A (BPA)), polychlorinated biphenyls (PCBs), and pesticides, as well as phytoestrogens naturally occurring in the environment.

### 1.2. Adrenal Incidentalomas

Adrenal incidentalomas have been detected in imaging tests performed for reasons other than a suspected adrenal pathology [8,9,10]. The literature data determining the frequency of AIs are divergent—it is estimated that AIs are detected in about 0.4% of patients undergoing abdominal US and in 0.6–2.9% of patients undergoing computed tomography (CT) [11,12]. Above all, the widespread use of chest computed tomography (CT) due to the COVID-19 pandemic has contributed to frequent incidental detection of adrenal lesions. Most AIs are asymptomatic benign lesions that do not reveal excessive hormonal activity [13]. It should be emphasized, however, that 2–4% of patients with AIs are diagnosed with adrenal cancer. The risk of cancer increases with the size of the tumor. When the diameter of the lesion exceeds 6 cm, the risk of malignancy increases up to 25%. The literature data reveal that the occurrence of AI in women is 2.5 times more frequent in life-related studies. Interestingly, in post mortem examinations, there were no such differences noticed [8,14]. These findings are probably related to imaging examinations when compared to men.

In addition to primary adrenal cancers, incidentally detected adrenal growths may also be metastatic—most commonly, kidney, lung and liver cancers. AIs may also be of inflammatory or granulomatous origin (in the course of tuberculosis or histoplasmosis), as well as hematomas, cysts, lipomas, liposarcomas, schwannomas or may occur in patients with congenital adrenal hyperplasia. On the other hand, some of the adrenal tumors originating from the adrenal gland may exert hormonal overactivity, presenting with glucocorticoid (Cushing’s syndrome), mineralocorticoid (Conn’s syndrome) or catecholamine (pheochromocytoma) hypersecretion. In different series, the probability of adrenal masses being functional varies between 6% and 30%. Besides the abovementioned overt clinical syndromes, more studies report the so-called subclinical autonomous hormone hypersecretion, referring mainly to subclinical autonomous hypercortisolemia and hyperaldosteronism. For this reason, the incidental detection of an adrenal mass requires regular, long-term hormonal and imaging control.

## 2. Common Relationships between the Thyroid and Adrenal Glands

Common hormonal relationships and their regulatory mechanisms between the thyroid and the adrenal glands are widely known. For example, numerous studies have shown that glucocorticoid (GC) excess has a suppressive effect on the hypothalamic–pituitary–thyroid (HPT) axis [15]. GCs also inhibit the peripheral conversion of fT4 to fT3 [16]. Animal studies have shown that thyroid hormones, via thyroid hormone receptor beta 1 (THRB1), which is present on fetal adrenal cortex cells, have a direct impact on their development, differentiation and function [17]. Similarly, both hyperthyroidism and hypothyroidism influence the hypothalamic–pituitary–adrenal (HPA) axis. An excess of thyroid hormones activates the HPA axis, whereas persistent thyroid hormone deficiency is associated with suppression of the HPA axis and a decrease in the adrenal secretion of cortisol [18,19,20]. Thyroid hormones regulate the function of the adrenal cortex also by influencing the hepatic metabolism of GCs. The research on animal models has shown that the deficiency of iodine, which is essential for thyroid hormone production, also inhibits the activity of the HPA axis, disturbs the circadian rhythm of cortisol and reduces its secretion in response to stress [15]. Thyroid dysfunctions also affect the renin–angiotensin–aldosterone (RAA) system [21]. It has been shown that the appropriate intracellular environment conditioned by thyroid hormones is necessary to achieve the full biological effect of catecholamine hormones produced by the adrenal medulla [22]. 

## 3. Insulin Resistance

Insulin resistance (IR) is a metabolic disorder characterized by a decreased sensitivity of cells and tissues to insulin. As a result, the metabolic effects of this hormone, mainly referring to carbohydrate, lipid and protein metabolism, are limited. This leads to a compensatory increase in insulin secretion and hyperinsulinemia [23,24]. However, with time, the compensatory mechanisms are insufficient to maintain the euglycemic state. Chronic hyperglycemia and the excess of free fatty acids in the mitochondria lead to the formation of free oxygen radicals and glucolipotoxicity. Therefore, IR is associated with oxidative stress and a low-grade chronic inflammatory state [25].

The model most commonly used in clinical practice is Homeostasis Model Assessment—Insulin Resistance (HOMA-IR) calculated based on the following mathematical formula: HOMA–IR (mmol/L × uU/mL) = fasting glycemia (mmol/L) × fasting insulinemia (mU/mL)/22.5.

It is assumed that, for physiological conditions, HOMA-IR should be below 1 [23,24]. However, the HOMA-IR cut-off point, above which insulin resistance is defined, has not been clearly determined, and values ranging from 1 to 2.55 are quoted in the literature [26]. 

### 3.1. Insulin Resistance and the Thyroid

The presence of both insulin and IGF1 receptors has been detected on the surface of human thyroid follicular cells [27]. Accumulating evidence suggests the interdependence between G-protein-coupled receptors, such as the TSH receptor (TSH-R), and tyrosine-kinase-coupled receptors. It has been proven in animal models that TSH, through cAMP, induces insulin receptor expression on the surface of thyrocytes and activates the insulin/IGF-1 signaling pathways in thyroid cells [28]. Significant research into thyroid TSH-R and IGF1-R crosstalk has recently been performed, and this crosstalk has proven to be one of the underlying causes of TSH-R-dependent diseases—Graves’ disease (GD) and Graves’ orbitopathy (GO) [29]. Interestingly, in 2020, an IGF1-R antagonist was officially registered by the Food and Drug Administration (FDA) for the treatment of active GO [30]. Moreover, it was proven, in one study, that IGF-1 stimulates the synthesis of specific cellular proteins that promote thyrocyte growth and differentiation. Furthermore, in vitro studies on thyrocyte lines derived from thyroid adenomas have shown that these cells can synthesize IGF-1 on their own. As a result, their growth can be stimulated in an autocrine or paracrine manner, leading to focal cell hyperplasia [31,32,33]. 

The discussed literature data prove that the insulin/IGF system plays an important role in regulating the growth and function of the thyroid gland. However, it is noteworthy that all of the components of the system are present not only in thyroid cells, but also in fetal and mature adrenal cell lines, and similarly, insulin seems to be a factor involved in their growth and differentiation [34,35,36].

Recently, an intriguing area of research has started focusing on the relationship between glucose metabolism disturbances and their effects on the endocrine system. Interestingly, Kalra et al. even proposed the term ‘glucocrinology’ to define the study of medicine that relates to endocrinopathies frequently coexisting with diabetes, hyperinsulinemia or metabolic syndrome [37]. For example, there is a growing body of data indicating that abdominal obesity and insulin resistance have a multifaceted impact on the function of the thyroid gland. It has been proven that obese people have higher TSH levels—in many studies, a positive correlation was observed between TSH and body mass index (BMI), fasting insulinemia and HOMA-IR [38,39]. In vitro studies have shown that the TSH receptor is present on the surface of human adipocytes [40]. Moreover, an increased TSH concentration can stimulate the proliferation of these fat cells and the increased release of adipokines and pro-inflammatory factors. The literature data have also revealed that TSH concentration is regulated, among others, by the hormones and neurotransmitters directly responsible for appetite control in the central nervous system, such as neuropeptide Y or leptin, which stimulate the release of TRH from the hypothalamus, thereby increasing TSH secretion by the pituitary gland [41]. These findings are consistent with the fact that metformin, a commonly known antihyperglycemic and insulin-sensitizing drug, lowers TSH levels in patients with overt or subclinical hypothyroidism. Interestingly, this effect has not been observed in euthyroid patients [42,43]. However, to date, no sufficient evidence has been collected that indicates these effects can be directly associated with an improvement in tissue insulin sensitivity. 

#### Insulin Resistance and Hashimoto’s Disease

The available literature also contains reports on the mutual relationships between obesity and insulin resistance with HD. Although this link is still unclear, it is noteworthy that each of these disorders is associated with a low-activity chronic inflammatory state and increased oxidative stress [44]. In a study by Liu et al., anti-TPO concentration was positively correlated with HOMA-IR and highly sensitive C-reactive protein (hsCRP), regardless of thyroid function [45]. In a study by Siemińska et al., higher concentrations of TSH and interleukin-6 (IL-6) have been demonstrated both in groups of patients with MS and in AITD patients [46]. In turn, Marzullo et al. determined that obese patients are more likely to have positive thyroid antibody titers, suggesting a potential relationship between leptin and autoimmunity [47]. Indeed, there are numerous theoretical premises that high plasma leptin, a hormone produced mainly in white adipose tissue, by activating effector T lymphocytes and inhibiting regulatory T lymphocytes, may favor the occurrence of autoimmunity in obese patients [48]. In a study by Waring et al., the authors showed a higher frequency of MS in patients with HD [49]. Another study showed a positive correlation between anti-TPO titers and body weight in women [50]. 

### 3.2. Insulin Resistance and Adrenal Incidentalomas

In recent years, extensive research has also been conducted to determine the factors related to the occurrence of adrenal incidentalomas. Among others, a higher frequency of insulin resistance was observed in patients with AIs [51]. So far, it has not been clearly established whether AIs develop because of pre-existing insulin resistance and are its clinical manifestation or whether insulin resistance is secondary to subclinical hypercortisolemia caused by the presence of AIs, undetectable by standard diagnostic methods [52,53]. Nevertheless, in the literature, we can find some clinical evidence of improvements in insulin sensitivity after the resection of a non-functional adrenocortical adenoma [54]. In addition, it has been widely proven that arterial hypertension, abdominal obesity and carbohydrate and lipid metabolism disturbances, as well as the diagnosis of MS, are more frequent in patients with AIs [55,56].

In light of the presented literature data, is it possible that insulin resistance and hyperinsulinemia may be among the factors contributing to the coexistence of HD and AIs? In a study by Karakose et al., patients with AIs had higher HOMA-IR, hs-CRP and ft4 levels and anti-TPO and anti-TG titers than the control group [57]. Moreover, in the group of AI patients, a positive correlation was observed between HOMA-IR, hs-CR and ft4 and the volume of the thyroid. These observations led the authors to hypothesize that seemingly inactive adrenal cortex adenomas may, in fact, produce some steroid hormones that cannot be detected by routine diagnostic methods. The overproduction of these hormones may, in turn, lead to the development of insulin resistance and, consequently, higher levels of ft4 in this group of patients. Still, it remains unclear whether the higher titer of anti-thyroid antibodies observed in AI patients is just a random finding or whether the presence of AIs is a risk factor for the occurrence of thyroid autoimmunity.

## 4. Endocrine Disruptors

According to the definition of the Polish Society of Endocrinology, EDs are exogenous chemical substances, synthetic or natural, that occur in the environment or food and interfere with the endocrine system [6]. According to WHO, EDs are specific compounds that can cause adverse effects on human bodies, offspring or subpopulations [7]. EDs include various substances such as dioxins, phthalates, phenols (including the commonly known Bisphenol A (BPA)), polychlorinated biphenyls (PCBs), and pesticides, as well as phytoestrogens that naturally occur in the environment. These compounds can influence the functioning of the endocrine system in numerous ways, including the disruption of steroid hormone synthesis, the affinity for androgen, estrogen or progesterone receptors or even a decrease in the sensitivity of the target tissues to hormones [6]. These disturbances can lead to metabolic disorders, allergies and neoplasms. EDs can disturb each of the hypothalamic–pituitary–peripheral gland axes and interfere with various hormonal signaling pathways [6].

### 4.1. Endocrine Disruptors and Obesity/Insulin Resistance 

The data available in the literature indicate that intrauterine exposure to EDs may contribute to obesity, insulin resistance, dyslipidemia, thyroid dysfunctions, infertility or a cancerous process in adulthood [58]. Studies have revealed that EDs may not only lead to an increase in adipocyte number and size, but may also disrupt the hormonal regulation processes involved in the maturation of adipose tissue [59,60]. In 2017, Darbre proposed the idea of the existence of the vicious circle mechanism, in which EDs increase the amount of adipose tissue in the body. The more fat cells there are in the organism, the bigger the accumulation of EDs and their secretion into the bloodstream and, thus, the higher the plasma concentration [61]. High levels of EDs favor the accumulation of adipose tissue.

### 4.2. Endocrine Disruptors and Thyroid Autoimmunity

It is commonly known that multiple chemical compounds used in industry and agriculture have a negative effect on the thyroid gland; for instance, perchlorates can impair iodine uptake and thiocyanates may reduce the activity of thyroid peroxidase [62,63]. Studies have proven that EDs not only influence the thyroid gland directly, but also interfere with the entire hypothalamic–pituitary–thyroid axis [64]. Some substances and chemicals have been shown to reduce the hypothalamic secretion of thyroid-releasing hormone (TRH) or inhibit the release of TSH from the pituitary gland, and others may interfere with plasma proteins that bind to thyroid hormones (e.g., thyroxin binding globulin—TBG) [64]. Moreover, many EDs can inhibit the binding of thyroid hormones to membrane cell receptors, reducing their transmembrane transport or activation by local tissue deiodinases [65]. Finally, EDs can also interfere with the metabolism of thyroid hormones [66]. In a recent study, an attempt was made to assess whether the pesticides commonly used in the environment can inhibit thyroid peroxidase activity in vivo, as it was previously observed in in vitro studies [67]. In pregnant rats exposed to these chemicals, a decrease in serum thyroid hormone levels was observed, with an accompanying increase in TSH and the volume of the thyroid gland. Furthermore, the authors of this study investigated whether these hormonal disturbances resulting from maternal exposure to pesticides would affect the development of the nervous system in the offspring. Indeed, heterotopia-like changes were found in the brain tissue of newborn rats [67]. Noteworthily, the presence of similar malformations was previously observed in studies performed on the offspring of rats exposed to high doses of anti-thyroid drugs during pregnancy. These results suggest that excessive exposure to pesticides in the prenatal period, by inhibiting the activity of thyroid peroxidase and the associated reduction in the concentration of maternal thyroid hormones available to the fetus, may lead to disorders analogous to decompensated hypothyroidism during pregnancy. Another study on animal models revealed that the exposure of pregnant sheep to 500 µg/d of BPA was associated with a decrease in fT4 and TT3 levels [68]. These results are of high significance, as it is commonly known that the endocrine systems of sheep and humans are very similar (especially referring to the thyroid axis), as are the pregnancy duration and the structure of the placenta. Therefore, these findings suggest that possibly analogous deviations can also be observed in humans. This hypothesis was confirmed in a large Korean study, in which pregnant women were tested for urine concentration of phthalates and BPA, which indirectly reflected the level of exposure to these substances and their plasma concentration. The study revealed a positive correlation between BPA and TSH concentrations [69]. Moreover, the authors determined that the higher the serum concentration of some phthalates, the lower the maternal ft4. Another study indicated that iodine deficiency may further exacerbate the negative effects of overexposure to EDs [70]. Taking into account the presented data, it seems necessary to undertake every effort to decrease the potential sources of ED exposure among women in the reproductive period, especially during pregnancy and lactation. Furthermore, it is noteworthy that ensuring an adequate iodine supply may limit the severity of the negative effects of ED overexposure.

### 4.3. Endocrine Disruptors and Adrenal Incidentalomas

Research into the effects of EDs on the adrenal glands is very limited. For example, although exposure to BPA is known to be associated with the disruption of steroidogenesis in reproductive tissues, little is known about its effects on the adrenal gland. However, theoretical premises indicate that structural and anatomical features of the adrenals make them very susceptible to ED-related disturbances. These features include rich vascularization, a lipophilic cell membrane with a high content of polyunsaturated fatty acids and the presence of cytochrome P450 enzymes—the source of toxic metabolites and free radicals [71]. Indeed, the inhibition of the activity of enzymes and regulatory proteins involved in the steroidogenesis pathway by BPA has been reported in the literature. In studies of Medwid et al., the authors aimed to evaluate the effects of prenatal BPA exposure on adrenal steroidogenesis in offspring—pregnant mice were exposed to a diet containing 25 mg/kg BPA throughout pregnancy. It was observed that in the adult offspring of the research group, the weight of the adrenal glands was significantly higher than in the offspring of the control group, and this finding was independent of changes in the plasma ACTH concentration [72]. This finding suggests that BPA exposure has a direct effect on adrenal development. Taken together, the presented study provides novel insights into the long-term consequences of developmental BPA exposure on adrenal steroidogenesis. Moreover, BPA increased the concentration of the steroidogenic acute regulatory protein (StaR) in offspring, which was accompanied by a higher plasma level of corticosterone [72]. To verify the underlying mechanism of these findings, Medwid conducted another study on human fetal adrenocortical cells as an in vitro model system. The author observed that BPA increased StAR protein expression through an estrogen receptor (ER)-mediated pathway [73]. Furthermore, using the same in vitro model system, Medwid demonstrated that BPA increased both the cell number as well as protein levels of the three universal markers of proliferation [74]. These findings prove that exposure to BPA can negatively affect adrenal gland development and steroidogenesis in adult mouse offspring. The presented literature-derived data come mainly from studies on animal or in vitro models. Studies assessing the influence of EDs on human adrenal cells in vivo are still very limited or conducted on small research groups. A study by Eker et al. performed on 50 patients with AIs indicated that the plasma concentration of BPA in the research group was significantly higher than in the control group [75]. These results may confirm the previously presented theoretical premises that BPA, being a model ED, can modify the expression of certain genes and promote the proliferation and transformation of adrenal cells. 

## 5. Summary

In view of the presented literature-based data, it is possible that the recent increase in the incidence of thyroid autoimmunity and adrenal gland tumors, as well as the epidemic of obesity and coexisting insulin resistance in developed countries, may have a common denominator. An imbalanced diet rich in processed foods, low physical activity and chronic stress—all of these components—interfere with the proper functioning of the endocrine system. Possibly, there are other, so far undiscovered, factors or mechanisms responsible for the increasing occurrence of the above-presented disorders. Nowadays, we are exposed to a wide variety of substances that modify and impair the functioning of the hormonal system. They are commonly found in industrial and agricultural activities, soil, water, air, everyday objects, food and cosmetics. It seems that exposure to these substances may not only lead to hormonal disturbances, but also to metabolic or proliferative disorders. Perhaps HD and AIs, accompanied by obesity and insulin resistance, are clinical manifestations of earlier, or even prenatal, overexposure to divergent hormonally active substances that we have recently started to describe as EDs. However, it should be once more emphasized that the impact of EDs on the human body is difficult to interpret as EDs do not occur individually in the environment, which makes it impossible to conduct research only on one specific chemical or one dysfunction. Another limitation is the lack of well-defined time frames between ED exposure—often in the embryonic period or very early in life—and the onset of the above-presented disorders. Still, there are not enough data to prove a direct relationship between HD, Ais and obesity and endocrine disruption, and therefore, this review is based mostly on indirect associations.

More properly designed studies on a sufficient research group, with a well-defined time exposure to a specific ED, are of high significance from a public health point of view. New methodologies are to be implemented, such as the use of human adrenal cell lines, to examine the effects of toxins and investigate ED action mechanisms. Simultaneously, the search for methods limiting the use of EDs and minimizing exposure to these substances in everyday life is of high importance. This work is aimed at increasing awareness that EDs have a negative effect, not only on the thyroid or adrenal glands but also on the entire endocrine system, and broadening the understanding of human health.

## Data Availability

Not applicable.
